# Perceived Risk of Infection Linked to Changes in Comfort in Social Situations From Before to During the COVID-19 Pandemic

**DOI:** 10.3389/fpsyt.2021.678072

**Published:** 2021-08-13

**Authors:** Janine Stierand, Finn Luebber, Sören Krach, Frieder Michel Paulus, Lena Rademacher

**Affiliations:** ^1^Social Neuroscience Lab at the Translational Psychiatry Unit (TPU), Department of Psychiatry and Psychotherapy, University of Lübeck, Lübeck, Germany; ^2^Department of Rheumatology and Clinical Immunology, University of Lübeck, Lübeck, Germany

**Keywords:** COVID-19, pandemic, social distancing, risk perception, mental health, social interactions, disease avoidance

## Abstract

**Background:** Social lives have significantly changed since social distancing measures have been implemented to prevent the spread of the coronavirus disease 2019 (COVID-19). This study aimed to investigate how our appraisal of social situations changed during the pandemic.

**Methods:** In two online surveys, conducted in October 2019 and April 2020, 58 participants rated their personal level of comfort for sketches depicting social situations. Situations were separately categorized according to the risk of a possible COVID-19 infection and changes in ratings were analyzed by using a repeated measures ANOVA. Moreover, potential influencing factors on the change in ratings such as perceived infection risk and social factors like regular frequency and liking of social interactions were examined.

**Results:** There was a significant interaction (*p* < 0.001) between time of measurement and risk category. Comfort ratings of depicted situations with low and medium infection risk were higher during the second compared to the first survey period. Ratings of high-risk situations did not change significantly, although there was a tendency toward lower ratings during the pandemic. Multiple regression analyses showed that perceived probability of short-term infection could explain variance in the change of ratings of social situations with low- and medium risk, but not perceived probability of long-term infection or social factors.

**Conclusion:** The results suggest that the change of participant's appraisal of the social situations during the COVID-19 pandemic relates to perceived infection risk. Both, the risk associated with the specific scenario as well as the general belief of short-term infection risk were associated with change. This change predominantly manifested in greater thought of comfort during low and medium risk situations, which might give a sense of safety during the pandemic. The finding that high-risk social situations were not rated as uncomfortable as expected must be considered with regard to the young sample and may not be generalizable to other individuals. Further research is necessary to evaluate long-term effects on social interactions caused by global pandemics such as the COVID-19 pandemic.

## Introduction

Since the beginning of the Coronavirus disease (COVID-19) pandemic in early 2020, our social lives have significantly changed. In most countries, social distancing measures were set in place to prevent the virus from spreading (1)^1^. Common activities that were previously considered to be pleasant, like going to a party or concert, eating at a restaurant, or even hugging or standing close to someone, now pose a potential threat to one's own and the society's health and safety. While social distancing measures are a necessary and effective intervention to keep the transmission of SARS-CoV-2 under control (2, 3), they also come with negative side effects. Several studies investigated how stay-at-home orders and social distancing measures during the first wave of the pandemic in 2020 affected mental health: Most, but not all (4) studies found negative effects on mental health (5–7), including higher levels of depression, anxiety (8), stress and tension (9), greater health anxiety, financial worry, and loneliness (10). Dawel et al. (11) found in a representative Australian sample that COVID-19-related impairments in work, financials, and social functioning were associated with reduced psychological well-being, irrespective of potential or actual exposures to SARS-CoV-2.

Apart from these effects on mental health, little is known about the consequences of social distancing guidelines and rules for our perception and appraisal of social interactions. It can be assumed that the constant threat of a potential infection may have an effect on how people perceive situations in which they engage with other persons. Anecdotally, Koren [(12), April 17] wrote in The Atlantic^2^:

“Sometime in the past few months, as social-distancing measures tightened across the country, many of us […] discovered new, pandemic-specific tics. […] The sight of two people shaking hands. Someone touching their uncovered face. A group of people hanging out less than six feet apart. Mundane behaviors [people] would not have thought twice about previously now trigger sudden, visceral reactions—of discomfort or disgust, fear or indignation—whether they're occurring on-screen or in real life. It almost seems as if the response to the pandemic has somehow, quietly and without warning, rewired our brains.”

Koren proposed that the COVID-19 pandemic had “created a collective aversion to previously innocuous behaviors and settings.”

Several studies found that the perceived risk of getting infected with SARS-CoV-2 increased with the implementation of protective measures and public health messages (13, 14) and is associated with experience with the virus as well as local occurrences of SARS-CoV-2-infections (15, 16). A higher risk perception is associated with more protective behavior such as social distancing and hand washing (13, 17) and might in turn lead to a devaluation of social situations with an increased risk of infection. To this date, research on change in the appraisal of social interaction in the course of the COVID-19 pandemic has been scarce. Casoria et al. (18) conducted an online experiment in France during the first wave of the COVID-19 pandemic (March–June 2020). Among other tasks, participants rated the appropriateness of the behavior of a hypothetical person inviting friends over for dinner (norm-elicitation task) and answered questions concerning their own compliance with social distancing measures (changes in behavior). They found that reported behavior and norm perception were closely related to current social distancing rules. In a review on the regulation of interpersonal distances, Welsch et al. (19) hypothesized that social distancing rules might lead to larger interpersonal distance preferences that could persist even after the end of the pandemic. However, as far as we know, there has been no research on the change in perception of social interaction during the COVID-19 pandemic so far.

To close this gap in previous research, the current study aimed at investigating how our appraisal of social situations changed during the ongoing pandemic. To this end, we used data of an online survey which was obtained in October 2019 before the pandemic started in the context of another research project and repeated data collection during the first wave of the pandemic in April 2020 with the same subjects. This stimulus material consisted of sketches depicting different social situations which were assessed by an independent group of raters for their risk regarding possible infection with COVID-19. Participants were asked to rate how comfortable they would feel in these situations. We hypothesized that change in comfort would depend on the risk of infection as implied in the social interaction. Specifically, the reported comfort in high-risk situations should reduce contrary to situations with lower risk of infection. Furthermore, we explored how interindividual differences regarding the fear of infection, frequency and liking of social interactions were associated with a possible change in people's appraisal of social situations during the pandemic. Lastly, we aimed to investigate whether there was a connection between the change in comfort ratings and changes in mental health of participants during the pandemic.

## Methods

The study was approved by the ethics committee of the University of Lübeck (AZ 18-078) and all subjects gave informed consent before starting each survey.

### Participants

Initially, *N* = 171 participants took part in a preliminary study to evaluate stimulus material for a planned neuroimaging project on eating behavior in October 2019. Participants were recruited via the University of Lübeck's student mailing list, via a Facebook group of psychology students at the University of Lübeck and via notice boards at the universities of Lübeck and Frankfurt. Since the planned neuroimaging project will only include female subjects, only women were asked to participate in the online survey. As compensation for their participation, they were able to choose between joining a lottery for winning 20 € or receiving course credits (only applicable for psychology students). All participants who had entered their e-mail address in the first survey (*N* = 170) were contacted again in April 2020 during the first wave of the COVID-19 pandemic in Germany and asked to fill out the same survey again. Seventy participants completed it a second time. As the original preliminary study was intended as a one-time measurement only, there was no clear assignment of participants to the data. Using the demographic information (age, height, weight, subject studied, and semester), we were able to successfully and distinctly match 63 cases. Of these, five cases were excluded for the analyses because of a potential bias in the ratings—one person because she reported she had been positively tested for COVID-19 and four participants because they claimed they had rated the sketches with regard to the current pandemic (see below for details on these decisions).

Thus, the final sample consisted of 58 participants (56 female, 1 male, 1 diverse) aged 19–37 years (*M* = 23.3; *SD* = 3.81). Most of them (91.4%) were University students, the others worked in the university/university hospital context (3 physicians, 1 medical technical assistant, 1 research assistant).

Most participants (77.2%) did not know anyone personally who had been infected with SARS-CoV-2. 17.5% knew one or two infected people personally, and 5.3% knew three or more persons (maximum: 7). All participants indicated that they were complying with the social distancing rules (37.9%: rather agree; 62.1%: agree strongly) and 77.6% of them stated that they considered COVID-19 more dangerous than the common flu.

### Online Surveys

**Figure 1:**
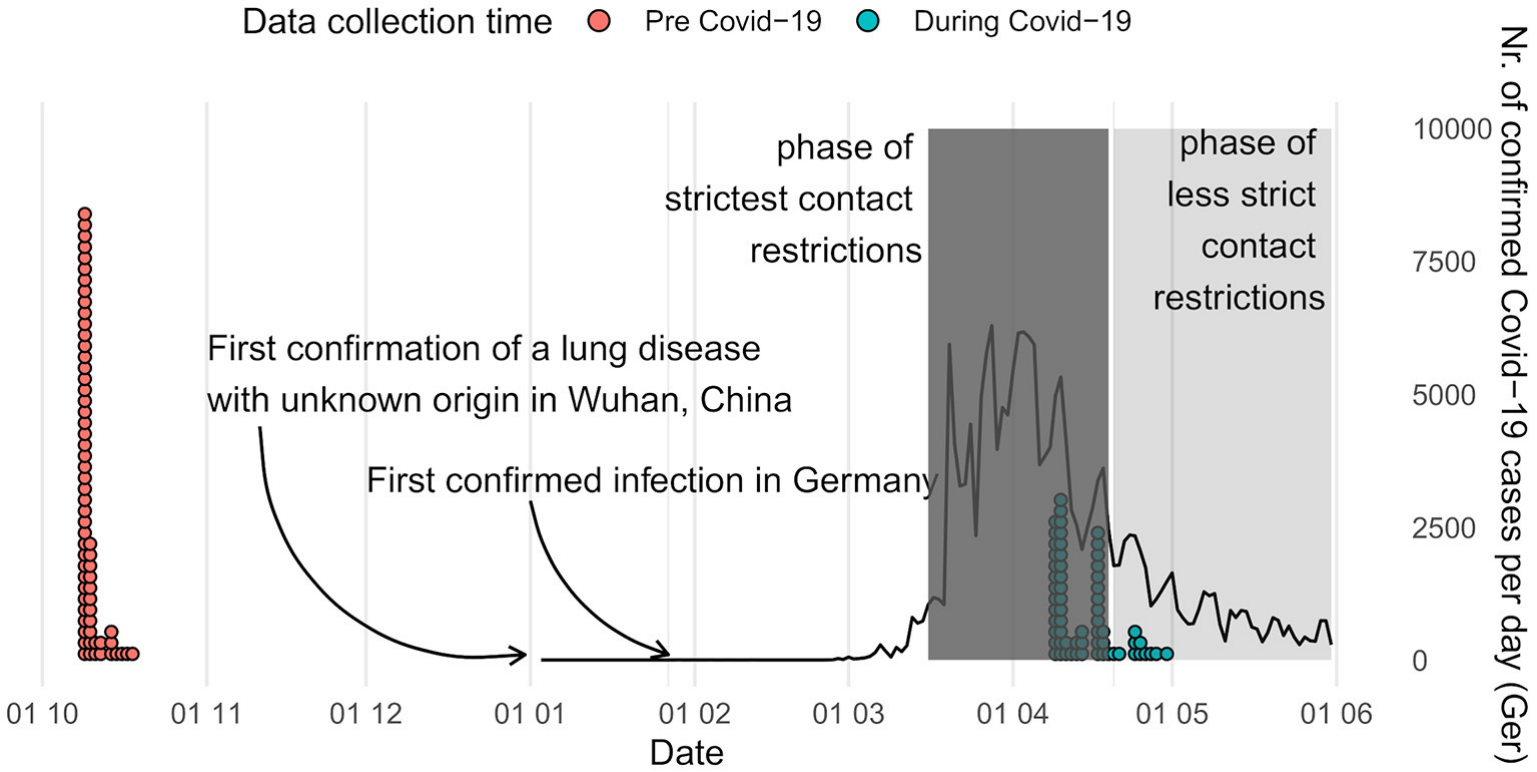
Time of data collection and overview of the development of the COVID-19 pandemic in Germany [(20); https://covid19.who.int/table]. Contact restrictions included cancellation of mass events, ban on gatherings of more than two persons, instructions to maintain a distance of more than 1.5 m to others, school and day care center closures as well as closures of all public spaces (e.g., playgrounds) and non-essential stores. Face masks in stores and public transport became mandatory on April 27.

The first online survey, which aimed at evaluating stimulus material for a planned fMRI project on eating behavior, was accessible on www.soscisurvey.com between October 9 and October 30 2019, i.e., long before the first headlines about COVID-19 went public (see Figure 1).

**Figure 2:**
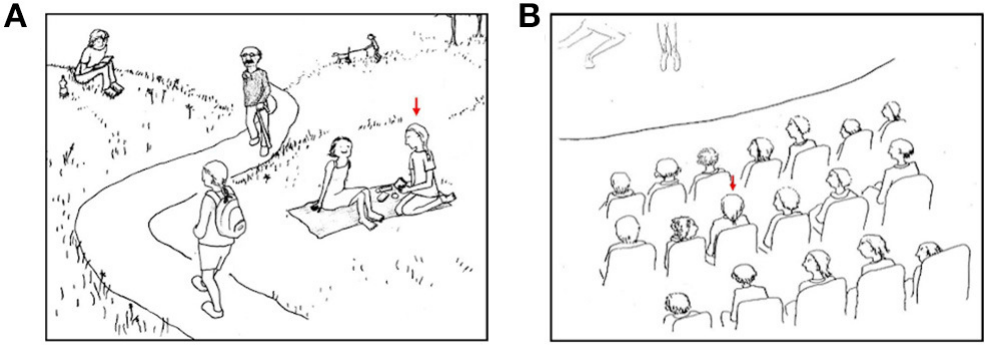
Two examples of sketches of situations related **(A)** and unrelated **(B)** to eating. Participants were asked to imagine being the person marked with a red arrow. These sketches were accompanied by the following descriptions: **(A)** “You are at a picnic with a friend. You are eating a sandwich.” **(B)** “You are at a performance. You are watching it intently”.

In the survey, participants were asked to rate 45 hand-drawn sketches of situations related or unrelated to eating according to how pleasant they found the situations (the stimulus material can be found under https://osf.io/xec9v/files/). Each sketch showed a different social situation with several persons (e.g., in the supermarket, restaurant, park, or on the street). Participants were asked to put themselves into the place of the person marked with a red arrow (see Figure 2). Each sketch was accompanied by a two-sentence description of the current situation (e.g., “You are on the train. You are looking for a seat.”). Participants were asked three questions about each sketch: (1) “How comfortable do you feel in this situation?”, (2) “How much do you feel like you're being observed in this situation?”, (3) “How ashamed does this situation make you feel?”. Responses were assessed on a scale ranging from one (e.g., “very uncomfortable”) to seven (“very comfortable”).

The second survey period extended from April 9 to April 30 during the first wave of COVID-19 infections and the period of the strictest social distancing measures in Germany up to that timepoint (see Figure 1). The goal of this second data collection was to examine changes in participants' evaluation of social situations after the beginning of the global pandemic. In order to avoid influencing the ratings and possibly creating a bias, the aim of the study was concealed from the participants before completing the survey. They were told that the purpose of the second survey was to examine the stability of the stimulus ratings over time. The first section of the survey was identical to the initial survey: Again, the same 45 sketches were presented. As described above, the sketches showed people in situations of varying social contexts and thus of varying risk of a possible COVID-19 infection. In order to stay consistent with the first survey, participants were asked the same three questions about each sketch, although only the first question (“How comfortable do you feel in this situation?”) was of interest in the context of the current study. After completing the sketch ratings, participants were informed about the true purpose of the current study. Afterwards, they were asked to answer questions related to the pandemic: They were asked how much they had thought of the current situation of social distancing while rating the pictures and then, more importantly, whether they rated the sketches in regard to the current situation during the pandemic or in a general sense, i.e., unrelated to the current circumstances. This question was asked because the current study aimed to capture differences in the perceptions of social situations that are not directly tied to the global pandemic (e.g., whether the situations violated social distancing measures). Four participants reported they had rated the sketches with regard to the current pandemic and were thus excluded from further analyses. Next, participants were asked questions about how they were being affected by the virus (e.g., “How many people do you know personally, who are or have been infected with the virus?”), estimates of the likelihood they might contract or transmit the virus within the next 2 weeks/2 months/year/in their lifetime [based on (21)], attitude toward restrictions/the virus (e.g., “I am following the current social distancing rules”), usual social habits and the liking thereof (e.g., “How often do you usually have physical contact with family members/friends/work colleagues/strangers […]”), fear of infection (e.g., “Are you worried about getting COVID?”), and quality of life (e.g., “How much have the pandemic and social distancing rules and their consequences affected you quality of life?”, for a full list of all questions see https://osf.io/xec9v/files/).

### Categorization of Sketches

Initially, the 45 sketches had been categorized into situations related and unrelated to eating. However, for the current study a different categorization was needed regarding the contact to other persons in the situation and the associated risk for a COVID-19 infection. To classify the sketches into different risk categories, 10 lab members independently rated the potential risk of an infection for each sketch as low, medium, or high. Sketches were assigned to one of the three risk categories if at least 50% of the raters had classified them in this category. Three sketches were excluded, because there was no majority of ratings for one risk category. To further explore the reasons for the subjective feeling of risk, each sketch was additionally rated with respect to several aspects of the situation (for a full list of questions see https://osf.io/xec9v/files/), for example compliance with social distancing rules (yes/no), sufficient area ventilation (yes/no), close physical contact (<1.5 m) for more than 15 min (estimated number of people), and risk of transmission by fomites (low/medium/high). The number of sketches classified into the three categories and the corresponding mean ratings of risk-associated aspects of the situations can be found in Table 1.

**Table 1:** Characteristics of the situations belonging to the three risk categories.

	**Risk category**		
	**Low risk**	**Medium risk**	**High risk**
Number of sketches	19	13	10
Compliance with social distancing rules (% Yes)	84.69	36.15	4.0
Ventilated area (% Yes)	89.47	27.69	11.0
Close physical contact (<1.5 m) for more than 15 min (number of persons)*, M* (*SD*)	0.321 (0.242)	0.842 (0.299)	3.02 (3.42)
Risk of transmission by fomites (1 = low, 2 = medium, 3 = high), *M* (*SD*)	1.25 (0.244)	1.87 (0.138)	2.15 (0.178)

### Data Analysis

Factor analyses were performed in R (22), all other statistical analyses were performed in Jamovi (23).

To get an impression of the impact the pandemic had on the participants, we first analyzed changes in quality of life and mental health using one-sample *t*-tests against the value of the respective scale which indicated no change (see https://osf.io/xec9v/files/ for details on the questions). Furthermore, we tested whether participants worried more about getting infected themselves or the infection of close other persons by using a paired-samples *t*-test.

Then, we tested our hypothesis about changes in the ratings of the social situations over time using a repeated measures ANOVA with the within-subject factors “time” (T1, T2) and “risk category” (low, medium, high). Mauchly's test indicated that the assumption of sphericity had been violated for the factor risk, therefore degrees of freedom were corrected using Huynh-Feldt estimates of sphericity (ε = 0.86). Correlations were used to evaluate the relationship between the above-mentioned mental health variables and changes in ratings over time.

Finally, we examined potential influences of the perceived probability of getting infected, the general frequency of social contacts, and the liking of social contacts on the change in the ratings of the situations of the different risk categories using multiple hierarchical regression. First, we reduced the number of covariates, because there had been several items to measure the perceived infection probability (within the next 2 weeks, 2 months, year, and in the participant's lifetime), the frequency of social contacts (with family members, friends, work colleagues, strangers at leisure activities, strangers while traveling, and strangers on commute to work or while doing essential shopping), and the liking of social contracts (with family members, friends, work colleagues, strangers at leisure activities, while traveling, and on commute to work or while doing essential shopping). Therefore, we first ran exploratory factor analyses (varimax rotation, factoring method maximum likelihood) using the “psych”-package for R (24) within the three aforementioned item blocks (infection probability, frequency of social contacts, and liking of social contacts). The number of factors were determined by parallel analysis, comparing the empirical eigenvalues to the 99th quantile of the simulated data. The four items about the perceived probability of getting infected yielded a two-factor solution. After varimax rotation (sum of squares loadings: 1.60, 1.41, proportion of variance explained: 0.40, 0.35), the items for perceived probability of getting infected within the next year and life-time loaded predominantly on one factor (loadings: 0.96, 0.70, respectively) and the infection probability for the next 2 weeks and 2 months on a second factor (loadings: 0.72, 0.90, respectively) with little cross-loadings between factors (<0.43). This suggests that people differ with respects to their perceived probability of getting acutely infected and the perceived probability of being infected in the long term. The six items about the frequency of social contacts yielded a one-factor solution (sum of squares loading: 1.98, 33% variance explained), regardless of the relationship to the other people. The factor structure of the six items about the liking of social contacts was more ambiguous but the parallel analysis favored a two factor solution, which we decided to use for further analyses. After varimax rotation (sum of squares loadings: 1.40, 1.16; proportion of variance explained: 0.23, 0.19), the two factors reflected individual differences in liking of meeting friends or people during leisure activities, and during traveling in one factor (loadings: 0.42, 0.99, 0.44; cross-loadings <0.29) and liking of meeting with family, work colleagues and commuting/shopping in another factor (loadings: 0.36, 0.86, 0.44; cross-loadings <0.14). Bartlett factor scores were extracted from all three factor analyses, resulting in five variables (two for perceived infection risk, one for frequency of social contacts, and two for liking of social contacts). These five variables were then used in the regression analyses to explain individual differences in change of comfort. Three individuals had to be excluded because they had not answered all questions regarding perceived infection risk, frequency or liking of social contacts, and factor scores could thus not be computed. Regression analyses were built for comfort ratings of each of the three risk categories separately using the following two-step procedure: The first model included confounding factors related to individual differences in social behavior (“social factors model”): (1) frequency of experiencing social interactions, (2) liking of work and family interaction, and (3) liking of social interactions during leisure time, holidays, and interactions with friends. The second model included the factors of interest related to perceived risk of infection (“infection risk factors model”): (4) perceived probability of short-term and (5) long-term infection risk. Model comparisons were conducted to compare the relative explanatory power between both sets of predictor variables.

## Results

### Reported Impact of the Pandemic on Quality of Life and Mental Health

Since the beginning of the pandemic participants had felt a decrease in their quality of life, deterioration of their mood, as well as an increase in tension and stress, while there was no significant change in anxiety, sleep, or alcohol consumption (see Table 2). A paired-samples *t*-test showed that participants were significantly more worried that persons close to them might get infected with SARS-CoV-2 (*M* = 3.245, *SD* = 0.96) than they were about their own possible infection [*M* = 2.26, *SD* = 0.93; *t*_(57)_ = −11.0, *p* < 0.001].

**Table 2:** Reported changes in quality of life and mental health since the beginning of the pandemic.

	***M* (*SD*)**	***t***	***df***	***p***
Quality of life	2.51 (0.69)	16.6	56	<0.001
Mood	3.51 (0.91)	4.23	56	<0.001
Tension and stress	3.46 (0.98)	3.50	56	<0.001
Anxiety	3.14 (0.61)	1.74	56	0.088
Sleep	3.14 (0.90)	1.18	56	0.242
Alcohol consumption	2.93 (0.78)	−0.64	56	0.498

*Responses were given on a 4-point Likert scale for quality of life (1 = not affected; 4 = very strongly affected) and a 5-point Likert scale for all other items (For mood and sleep: 1 = significantly improved; 3 = unchanged; 5 = significantly worsened. For tension and stress, anxiety, and alcohol consumption: 1 = significantly less; 3 = unchanged; 5 = significantly more)*.

### Changes in Ratings From Before to During the Pandemic

**Figure 3:**
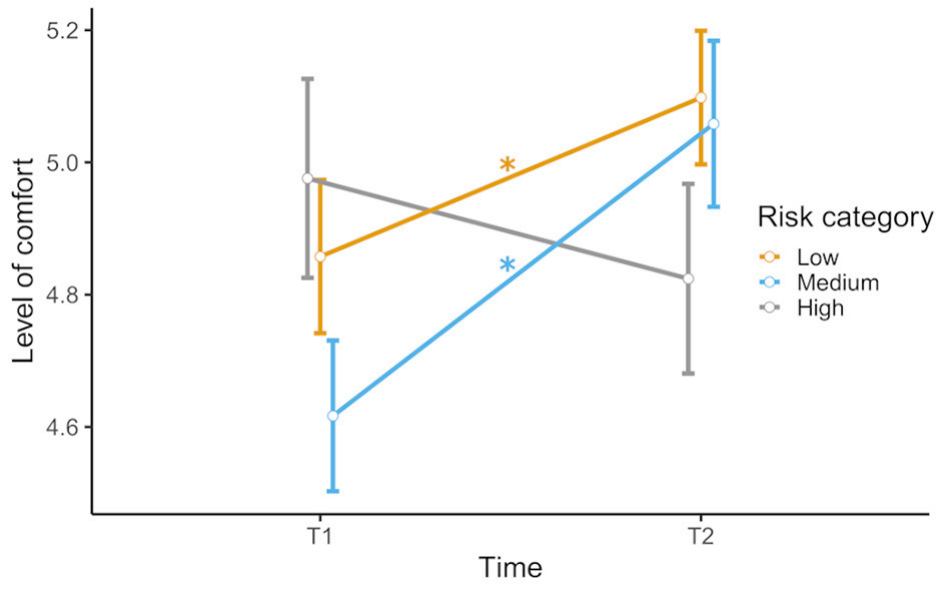
Rated level of comfort in low-, medium-, and high-risk scenarios before (T1) and during (T2) the COVID-19 pandemic. Error bars indicate standard error (S.E.). Significant comparisons of T1 and T2 are indicated by asterisks.

There was a significant main effect of the factor time [*F*_(1,57)_ = 5.82, *p* = 0.019, $\eta_{\text{p}}^{2}$ = 0.093], but no significant main effect of risk category [*F*_(1.72, 98.25)_ = 3.10, *p* = 0.057, $\eta_{\text{p}}^{2}$ = 0.052]. However, there was a significant interaction between time and risk category [*F*_(2,114)_ = 24.26, *p* < 0.001, $\eta_{\text{p}}^{2}$ = 0.299]. *Post-hoc t*-tests showed that situations of low and medium risk were rated significantly more comfortable in the second survey [low risk: *t*_(57)_ = −2.83, *p* = 0.006; medium risk: *t*_(57)_ = −4.91, *p* < 0.001], while ratings for situations of high risk did not differ significantly between the two surveys, *t*_(57)_ = 1.65, *p* = 0.105 (see Figure 3).

To analyze whether changes in ratings of the sketches were associated with reported changes in mental health, correlations were calculated. None of the variables mentioned above (quality of life, mood, tension/stress, anxiety, sleep, and alcohol consumption) correlated with changes in ratings (0.11 < *p* < 0.995).

### Influences of Individual Differences in Social Behavior and Perceived Probability of Infection on Change in Comfort

To assess the potential impact of individual differences on the change of the level of comfort in situations of the three different risk categories, we explored associations with individual frequency and liking of social interactions (social factors) as well as perceived probability of infection (infection risk factors) using model comparisons in multiple regressions. Results indicated that the social factors did not significantly explain variance in the change of comfort for neither risk category (see Table 3). However, the model which additionally included the infection risk factors had a significant increase in the explained variance for the low [ΔR^2^ = 0.12, *F*_(2,49)_ = 3.70, *p*_*adj*_ = 0.048] and medium risk situations [ΔR^2^ = 0.19, *F*_(2,49)_ = 5.93, *p*_*adj*_ = 0.015]. *Post-hoc* examinations of the individual predictors in the model showed that only the associations with the perceived short-term probability of infection were significant for both the low (*B* = 0.23, *SE* = 0.08, *t*_(49)_ = 2.693, *p*_*adj*_ = 0.015, β = 0.35, 95% CI: [0.09;0.62]) and medium-risk (*B* = 0.27, *SE* = 0.09, *t*_(49)_ = 3.20, *p*_*adj*_ = 0.006, β = 0.42, 95% CI: [0.16;0.68]) situations. For high-risk situations, the infection risk model did not explain variance in the change of comfort significantly (see Table 3). All *p*-values were corrected for multiple comparisons between the three models using the Benjamini-Hochberg-procedure (25), as implemented in the “stats”-package for R. For detailed model fit indices and estimates for individual predictors please see Supplementary Tables 1–3.

**Table 3:** Outcomes of multiple regression analyses for all three risk categories with differences in ratings of the sketches as outcomes and factor scores from factor analyses (of variables on perceived probability of infection, frequency of social contacts, and linking of social contacts) as regressors.

	**Social factors (M1)**			**Infection risk factors (M2)**		
	***F* (*df*)**	***p* (adj.)**	***ΔR2***	***F* (*df*)**	***p* (adj.)**	***ΔR2***
Low risk	1.30 (3, 51)	0.283 (0.849)	0.07	3.70 (2, 49)	0.032 (0.048)	0.12
Medium risk	0.11 (3, 51)	0.953 (0.953)	0.01	5.93 (2, 49)	0.005 (0.015)	0.19
High risk	0.25 (3, 51)	0.863 (0.953)	0.01	0.55 (2, 49)	0.581 (0.581)	0.02

*The “Social Factors” model served as baseline model and contained the three factor scores representing individual differences in the general frequency of social interactions, liking of interaction at work and with family, liking of interactions with friends, and liking of social interaction during leisure time. The “Infection Risk Factors” model additionally included two variables representing the perceived short-term or long-term probability of infection with SARS-CoV-2. Adj.: adjusted p-value after Benjamini-Hochberg-correction for comparison between the three models*.

## Discussion

The COVID-19 pandemic and its ramifications have had a substantial impact on people's lives all over the world. The effects on mental health have been the object of several studies. Most studies found negative effects on mental health (5–7), including higher levels of depression, anxiety (8), stress and tension (9), greater health anxiety, financial worry, and loneliness (10). Here we extend on these findings and show how the threat of a SARS-CoV-2 infection and social distancing measures have affected people's perception and appraisal of social interactions from before to during the COVID-19 pandemic.

Our main finding is that comfort ratings of social situations changed from before to during the first wave of the COVID-19 pandemic and this change depended on the risk of infection. Importantly, this change in comfort could be explained by the perceived short-term risk of infection.

The general pattern well-reflects the prior assumption that change in the appraisal of social situations varies according to the inherent risk of infection and depicted violations of social distancing rules, however, our findings also deviate from initial anecdotal observations. While the rated level of comfort increased for low and medium risk situations, there was no statistically significant change in the ratings of high-risk situations, although there was a tendency toward a lower level of comfort during the pandemic. The negative appraisal of high-risk situations could not be confirmed by our findings. One possible explanation for these results might be that participants were asked to rate rather abstract drawn sketches of social situations, which might not be able to elicit as strong responses as real situations or seeing social interactions on TV. Furthermore, our sample consisted of young participants (all younger than 38 years) based in Northern Germany, an area in Germany that was not severely affected by the pandemic during the time of data acquisition. Even though they reported a negative effect of the pandemic or, more likely, the social distancing measures on their mental health, they were more worried about people close to them (presumably older relatives) getting infected than they were about themselves. Thus, imagining themselves in the portrayed situations may not have seemed too threatening as it might have been for people belonging to a high-risk group. Additionally, history effects could contribute to the observed differences. It is possible that the perceived comfort of social situations in such kind of thought experiment might increase in context of social deprivation. Such changes in the appraisal of social interactions should overlay the changes associated with infection risk and might generally attenuate ratings toward being more positive as they would fulfill their need for being socially integrated. Such confound would result in a similar response pattern as observed here with high-risk social situations not being evaluated as uncomfortable as initially hypothesized. The high-risk situations in our sketches consisted mainly of social gatherings such as parties and eating out at restaurants. Considering the fact that participants stated that they had rated the sketches in a general sense and not in regard to the current pandemic, the typical desirability of those kinds of situations might have outweighed the negative connotation during an ongoing pandemic.

The change in appraisal of social situations during the pandemic seems to be more apparent in low- to medium-risk social situations in our study. In this context it is important to note that we found the perceived probability of short-term infection, but not the frequency or liking of social contacts or the perceived probability of infection in the long term, to explain variance in the change of ratings of low- and medium risk social situations. The higher participants rated the probability of getting infected in the upcoming weeks the more comfortable low- and medium-risk situations were rated during the pandemic compared to before. These findings support the notion that pandemic-related cognitions affect the different appraisal of social scenarios. The increased motivation for and implementation of protective measures due to a heightened perceived risk of infection with SARS-CoV-2 (13, 17) possibly lead to a higher valence of lower risk social situations that are more in line with personal self-restraint intentions (14). It might thus be the case that lower risk situations were more appreciated during the first wave of the pandemic because they give a sense of safety. Policy makers should keep this mechanism in mind, as individual risk perception seems to play an important part in the adherence to social distancing measures. Thus, a clear communication by authorities which aspects of a situation are most important for viral transmission might increase compliance with preventive measures by influencing risk perception and (dis-)liking of situations with these characteristics.

Similar to previous findings (5–7, 9), participants reported a decrease in their quality of life and a deterioration of mood, as well as an increase in tension and stress and a tendency toward increased anxiety. There was no significant change in quality of sleep or alcohol consumption. None of these variables correlated with changes in ratings of the depicted social situations.

To our knowledge, this study is the first examining the change of people's appraisal of social situations during the COVID-19 pandemic. One of its strengths is that there was data available collected before the pandemic which could be compared to data collected from the same subjects during the first wave of the pandemic. However, this advantage also comes with the limitation that the sketch material was originally designed for another purpose. Thus, risk categories had to be defined retrospectively and comprised a varying number of sketches. Also, there was no data on participant's mental health in the first survey and participants were asked about changes in their mental health retrospectively with one item questions per category (i.e., depression, anxiety, etc.) in the second survey. This limits the reliability and validity of these measures. This study also has several other limitations. First, the sample consisted of relatively young, mostly female, and highly educated participants and is therefore not representative for the German population. Presumably, effects would have been significantly stronger in a high-risk sample. It should also be noted that the low number of responses in the second survey (70 compared to 170) could indicate a selection bias, for example those subjects most affected by the pandemic might not have responded to the request for the second survey. Second, data was only collected during the first wave of the pandemic from March until May 2020. It is thus unknown if or how effects change over the course of the pandemic and how long they may persist after the pandemic. The findings of Casoria et al. (18) suggest that the appraisal of social situations is closely related to current social distancing measures and may thus change back to normal once the pandemic is over. However, another possible development is that this pandemic will have long-lasting effects on how people will interact with each other in the future, possibly always keeping the threat of another infectious disease in mind, as proposed by Welsch et al. (19). Long-term studies will be needed to further evaluate possible lasting effects on social interactions caused by pandemic events.

## Data Availability Statement

The datasets of the two online surveys are not readily available because no consent had been obtained from the participants since the study was originally planned only as a pilot study of another project. Requests to access data should be directed to the corresponding author. Datasets of the ratings for the categorization of sketches into risk categories can be found under https://osf.io/xec9v/files/.

## Ethics Statement

The studies involving human participants were reviewed and approved by Ethics Committee of the University of Lübeck. The participants provided their written informed consent to participate in this study.

## Author Contributions

LR, SK, and JS conceived the research and designed the questionnaire. JS, LR, FL, and FP performed data analysis and wrote the manuscript. SK revised the manuscript. All authors discussed the results, contributed to the article, and approved the submitted version.

## Conflict of Interest

The authors declare that the research was conducted in the absence of any commercial or financial relationships that could be construed as a potential conflict of interest.

## Publisher's Note

All claims expressed in this article are solely those of the authors and do not necessarily represent those of their affiliated organizations, or those of the publisher, the editors and the reviewers. Any product that may be evaluated in this article, or claim that may be made by its manufacturer, is not guaranteed or endorsed by the publisher.

## Footnotes

^1^https://www.who.int/publications/i/item/overview-of-public-health-and-social-measures-in-the-context-of-covid-19.

^2^https://www.theatlantic.com/science/archive/2020/04/coronavirus-pandemic-cringe/610180/.
