# Supplementary material for: Perceived Risk of Infection Linked to Changes in Comfort in Social Situations From Before to During the COVID-19 Pandemic
Source: Front Psychiatry. 2021 Aug 13;12:678072. doi: 10.3389/fpsyt.2021.678072 (PMC8415019; doi:10.3389/fpsyt.2021.678072)
Supplement: Supplementary file 1 [file Data_Sheet_1.docx]

Supplementary Material

Table S1: Model fit measures for Model 1 with only social factors (baseline model) and Model 2 with social factors and infection-related factors

|  | **Low risk** | **Medium risk** | **High risk** |
| --- | --- | --- | --- |
| **Model 1** |  |  |  |
| *R^2^* | .07 | .01 | .01 |
| *F*(3,51) | 1.30 | 0.11 | 0.25 |
| *p* (adj.) | .283 (.849) | .953 (.953) | .863 (.953) |
| AIC | 113 | 119 | 126 |
| **Model 2** |  |  |  |
| *R^2^* | .19 | .20 | .04 |
| *F*(5,49) | 2.34 | 2.45 | 0.36 |
| *p* (adj.) | .055 (.083) | .046 (.083) | .870 (.870) |
| AIC | 109 | 111 | 129 |

*Notes.* AIC: Akaike information criterion. Adj.: adjusted *p*-value after Benjamini-Hochberg-correction for comparison between the three models

Table S2: Individual statistics for the coefficients of the baseline model for all three risk categories

|  | **Low risk** | **Medium risk** | **High risk** |
| --- | --- | --- | --- |
| **Intercept** |  |  |  |
| *B* (*SE*) | 0.23 (0.09) | 0.43 (0.09) | -0.17 (0.10) |
| *β[CI]* | - | - | - |
| *t* | 2.61 | 4.66 | -1.72 |
| *p* (adj.) | .012 (.018) | < .001 (< .001) | .092 (.092) |
| **Frequency contact** |  |  |  |
| *B* (*SE*) | 0.24 (0.13) | 0.00 (0.13) | 0.09 (0.14) |
| *β[CI]* | .32 [-.02; .66] | .00 [-.35; .35] | .11 [-.245; .456] |
| *t* | 1.89 | 0.01 | 0.61 |
| *p* (adj.) | .065 (.195) | .995 (.995) | .547 (.821) |
| **Liking contact work & family** |  |  |  |
| *B* (*SE*) | -0.05 (0.10) | -0.03 (0.11) | -0.02 (0.11) |
| *β[CI]* | -.07 [-.39; .24] | -.04 [-.37; .28] | -.03 [-.35; .30] |
| *t* | -0.47 | -0.26 | -0.18 |
| *p* (adj.) | .638 (.856) | .793 (.856) | .856 (.856) |
| **Liking contact friends & leisure** |  |  |  |
| *B* (*SE*) | -0.11 (0.11) | 0.05 (0.12) | -0.10 (0.13) |
| *β[CI]* | -.14 [-.44; .16] | .07 [-.24; .38] | -.12 [-.43; .19] |
| *t* | -0.94 | 0.44 | -0.80 |
| *p* (adj.) | .352 (.645) | .665 (.665) | .430 (.645) |

*Notes.* B: unstandardized predictor, SE: standard error of the mean, β: standardized predictor, CI = 95% confidence interval, df for individual predictors: 51. Adj.: adjusted *p*-value after Benjamini-Hochberg-correction for comparison between the three models

Table S3: Individual statistics for the coefficients of the full model for all three risk categories

|  | **Low risk** | **Medium risk** | **High risk** |
| --- | --- | --- | --- |
| **Intercept** |  |  |  |
| *B* (*SE*) | 0.24 (0.08) | 0.44 (0.08) | -0.17 (0.10) |
| *β[CI]* | - | - | - |
| *t* | 2.86 | 5.21 | -1.68 |
| *p* (adj.) | .006 (.009) | < .001 (< .001) | .099 (.099) |
| **Frequency contact** |  |  |  |
| *B* (*SE*) | 0.27 (0.12) | 0.05 (0.12) | 0.11 (0.15) |
| *β[CI]* | .36 [.03; .69] | .07 [-.26; .40] | .14 [-.22; .50] |
| *t* | 2.22 | 0.43 | 0.76 |
| *p* (adj.) | 0.031 (.093) | .668 (.668) | .451 (.668) |
| **Liking contact work & family** |  |  |  |
| *B* (*SE*) | -0.02 (0.10) | 0.00 (0.01) | -0.02 (0.12) |
| *β[CI]* | -.03 [-.33; .27] | .00 [-.30; .31] | -.02 [-.35; .31] |
| *t* | -0.20 | 0.03 | -0.14 |
| *p* (adj.) | .840 (.976) | .976 (.976) | .892 (.976) |
| **Liking contact friends & leisure** |  |  |  |
| *B* (*SE*) | -0.13 (0.11) | 0.03 (0.11) | -0.10 (0.13) |
| *β[CI]* | -.17 [-.46; .11] | .03 [-.25; .32] | -.13 [-.44; .19] |
| *t* | -1.21 | 0.23 | -0.81 |
| *p* (adj.) | .231 (.630) | .819 (.819) | .420 (.630) |
| **Perceived infection probability (long term)** |  |  |  |
| *B* (*SE*) | 0.03 (0.09) | 0.11 (0.09) | 0.08 (0.10) |
| *β[CI]* | .05 [-.21; .31] | .17 [-.10; .43] | .12 [-.17; .40] |
| *t* | 0.38 | 1.27 | 0.82 |
| *p* (adj.) | .704 (.704) | .210 (.624) | .416 (.624) |
| **Perceived infection probability (short term)** |  |  |  |
| *B* (*SE*) | 0.23 (0.08) | 0.27 (0.09) | 0.07 (0.10) |
| *β[CI]* | .35 [.09; .62] | .42 [.16; .68] | .09 [-.19; .38] |
| *t* | 2.69 | 3.20 | 0.65 |
| *p* (adj.) | .010 (.015) | .002 (.006) | .516 (.516) |

*Notes*. B: unstandardized predictor, SE: standard error of the mean, β: standardized predictor, CI = 95% confidence interval, df for individual predictors: 49. Adj.: adjusted *p*-value after Benjamini-Hochberg-correction for comparison between the three models
